# Strategies and cost-effectiveness evaluation of persistent albuminuria screening among high-risk population of chronic kidney disease

**DOI:** 10.1186/s12882-017-0538-1

**Published:** 2017-04-18

**Authors:** Huaiyu Wang, Li Yang, Fang Wang, Luxia Zhang

**Affiliations:** 1 Renal Division, Department of Medicine, Peking University First Hospital; Peking University Institute of Nephrology, 8 Xishiku Street, Xicheng District, Beijing, 100034 China; 2 Department of Health Policy & Management, School of Public Health, Peking University Health Science Center, Beijing, China

**Keywords:** Persistent albuminuria, Screening strategy, Chronic kidney disease, Cost-effectiveness analysis

## Abstract

**Background:**

Screening for persistent albuminuria among the high-risk population is important for early detection of CKD while studies regarding screening protocol and related cost-effectiveness analysis are limited. This study explored a feasible and cost-efficient screening strategy for detecting persistent albuminuria among the high-risk population.

**Methods:**

A cohort study including 157 clinically stable outpatients with a risk factor of CKD and whose laboratory tests revealed an albumin-creatinine-ratio (ACR) between 30 and 300 mg/g of creatinine during the previous 12 months was conducted to assess the validity of alternative screening strategies. Each participant collected three first morning urine samples in three consecutive months. These samples were labeled as DAY-1, MONTH-2 and MONTH-3. In the first month, a random spot sample in the afternoon of the first day and another morning sample on the second day were collected and labeled as Random and DAY-2. Persistent albuminuria was defined by abnormal ACR (≥30 mg/g creatinine) for DAY-1, MONTH-2 and MONTH-3. Alternative strategies were DAY-1; Random; DAY-1 + Random; DAY-1 + DAY-2; and DAY-1 + Random + DAY-2. To evaluate the economic performance of those alternative strategies, a hybrid decision tree/Markov model was developed based on the cohort study to simulate both clinical and cost-effectiveness outcomes. Sensitivity analyses were conducted to investigate assumptions of the model and to examine the model’s robustness.

**Results:**

Altogether, 82 patients exhibited persistent albuminuria. All of the five strategies had sensitivity higher than 90%. Of these strategies, Random had the lowest specificity (46.7%), and DAY-1 + Random + DAY-2 had the highest specificity (81.3%). Estimated cost for each quality adjusted life year (QALYs) gained were ¥112,335.88 for DAY-1 + Random, ¥8134.69 for Random and ¥10,327.99 for DAY-1 + Random + DAY-2. When compared with DAY-1 strategy, the sensitivity and specificity of which were 100.0 and 69.3%, respectively. DAY-1 + Random + DAY-2 had the highest effectiveness and incremental effectiveness of 11.87 and 0.73 QALYs. At a willingness-to-pay threshold of ¥100,000 per QALY, DAY-1 + Random + DAY-2 had the highest acceptability of 91.0%. Sensitivity analyses demonstrated the robustness of the results.

**Conclusions:**

In order to make a quick diagnosis of persistent albuminuria among high-risk population, the strategy of combining two first morning urine samples and one randomized spot urine sample in two consecutive days is accurate, saves time, and is cost-effective.

## Background

Chronic Kidney Disease (CKD) is a worldwide public health problem associated with multiple adverse outcomes and a heavy burden on the healthcare system [1, 2]. Early diagnosis and intervention have been demonstrated to be effective to improve prognosis and reduce the burden of CKD [3, 4]. Among indicators of kidney damage, albuminuria has been proven to be an independent risk factor of end-stage renal disease, cardiovascular disease, and mortality in the CKD population. Hence, the current clinical guidelines advocate that persistent albuminuria (lasting for 3 months or longer) is an essential criterion for CKD diagnosis [2, 5–7].

However, most of the current large-scaled studies were based on single-measurement of albuminuria [8–11]. Previous studies show that the fluctuation of albuminuria is substantial. The repetition positive rate based on an initial positive measurement is only 50% in CKD 1–2 stage [12, 13]. The day-to-day variations of normo- and micro-albuminuria are ±467 and ±170%, respectively [14]. Therefore, relying on a single measurement of albuminuria might lead to over-estimation of disease prevalence, as well as unnecessary treatments. The CKD screening process should start with an accurate identification of persistent albuminuria.

Previous studies had investigated the population, testing items, and frequency of CKD screening [2, 9, 15, 16]. Cost-effectiveness analyses showed that it is more effective in screening high risk populations including diabetes, hypertension, and coronary heart disease, as compared to the general population [9, 15, 16]. Patients who had been diagnosed as persistent mild-to-moderate albuminuria or early stage CKD were recommended to re-assess ACR and eGFR once a year, but few of the previous studies explored or evaluated the screening protocol for persistent albuminuria [2].

Current clinical guidelines suggest that single-positive albuminuria results should be rechecked twice in the following two months to identify whether it is persistent [2]. However, for practical reasons, the time period of 2 months is not ideal in both clinical and research settings. A short time period of diagnosis is essential for maintaining a satisfactory response rate in both clinical and research practices. Developing a practical, time-saving, and cost-effective screening strategy with acceptable accuracy is definitely necessary.

This cohort study was conducted to establish such an initial, accurate, and time-saving screening strategy. Using the results of this study, a cost-effectiveness evaluation was completed. The purpose was to evaluate its economic performance from a societal perspective.

## Methods

### Participants

In the cohort study, outpatients admitted to Peking University First Hospital from January 2013 to January 2014, and who met the following inclusion criteria were included: 1) laboratory tests revealed an elevated ACR (30-300 mg/g creatinine) during the previous 12 months; 2) prior diagnosis of hypertension, diabetes, or coronary heart disease, but without pre-diagnosed kidney disease (including polycystic kidney disease, kidney tumor, transplanted kidney, primary or secondary glomerulonephropathy, renal tubular interstitial disease, acute kidney injury, and CKD). Exclusion criteria included: age younger than 45 years; urinary tract infection; being hospitalized within the past 2 weeks; being pregnant or less than 6 months postpartum; congestive heart failure; receiving chemotherapy and/or radiotherapy in the past 12 months. Altogether 160 patients who met the inclusion criteria were willing to participate. This group did not meet any of the exclusion criteria.

Each participant received face-to-face training and an instruction book on sample collecting. All subjects completed questionnaires to document their socio-demographic status, personal/family medical history (e.g., hypertension, diabetes, and kidney disease) and lifestyle (e.g., smoking, exercise) with assistance from nurses familiar with the study. A history of medications used (e.g., nephrotoxic medications, angiotensin-converting enzyme inhibitors, angiotensin receptor blockers, and statins) was also obtained.

In the subsequent cost-effectiveness study, the target population was patients with high risk factors of CKD including diabetes, hypertension, and coronary heart disease. It is well recognized that this population possesses higher detectable rates of persistent albuminuria and it is also the target population for CKD screening [9, 15–17]. The starting age was 45 years which is the threshold of higher prevalence of chronic diseases including diabetes, hypertension, and coronary heart disease as well as CKD, reflecting the commonly accepted starting age of screening for persistent albuminuria [18].

### Specimens and measurements

**Fig. 1:**
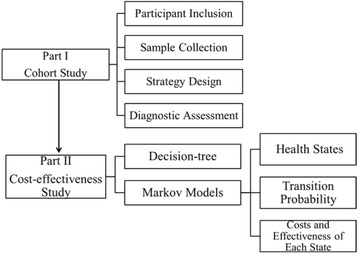
Flow Diagram. A cohort study was conducted initially to design and evaluate the alternative strategies for persistent albuminuria diagnosis. Participants aged over 45 years with a previous diagnosis of diabetes, hypertension, and/or coronary artery disease were included. Cost-effectiveness analysis was conducted based on the results of the cohort. Target population of the cost-effectiveness analysis was high risk population of CKD. A hybrid decision tree and Markov models were constructed to simulate the clinical pathway and long-term outcome

**Fig. 2:**
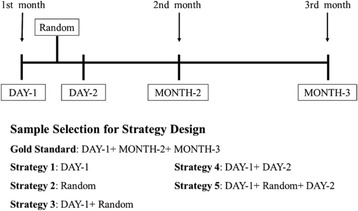
Sample Collection Flow Diagram and Strategy Design. First morning urine samples were collected 3 times (in 3 consecutive months), labeled as DAY-1, MONTH-2 and MONTH-3, respectively. A randomized spot urine sample was collected in the first day afternoon in the first month, labeled as Random. One more first morning urine sample was collected on the second day in the first month, labeled as DAY-2. Positive test for abnormal ACR was defined as ACR ≥ 30 mg/g creatinine

The complete process of the present study including Part I Cohort Study and Part II Cost-effectiveness Study is shown in Fig. 1. The process of sample collection and strategy design is shown in Fig. 2. Each participant was asked to collect five urine samples in three consecutive months. Three first morning urine samples on the same day in three consecutive months were collected and labeled as DAY-1, MONTH-2 and MONTH-3, respectively. Because of the day-to-day variations of albuminuria, one additional first morning urine sample on the second day in the first month was required, which was labeled as DAY-2. Due to the intraday variations caused by factors such as hydration status and physical activity, a random spot urine sample in the afternoon of the first day was required, which was labeled as Random (Fig. 2). In the first month, these two first morning samples and one randomized spot sample were to be stored in 4 °C refrigerators until participants sent them to the hospital on the morning of the second day. All the samples were cryopreserved in the same −80 °C refrigerator. The same test item was completed within two consecutive batches in order to avoid biases caused by the test itself.

Albuminuria was measured by immunoturbidimetic methods. Urinary creatinine was measured by Jaffe’s method on a Hitachi 7170 autoanalyzer (Hitachi, Tokyo, Japan). Albumin to creatinine ratio (ACR; mg/g creatinine) was calculated. A positive result was defined as an ACR greater than 30 mg/g creatinine. The term of “albuminuria” was used to describe the presence of either microalbuminuria or macroalbuminuria.

For eGFR, overnight fasting blood samples were collected by venipuncture on interview days in the first and the third months. Serum creatinine (SCr) was measured by Jaffe’s method on a Hitachi 7170 autoanalyzer (Hitachi, Tokyo, Japan). Estimated GFR was calculated using an equation developed by an adaption of the Modification of Diet in Renal Disease (MDRD) study based on data of Chinese CKD patients [19].

$$ \mathrm{eGFR} = 175\times {\mathrm{SCr}}^{\hbox{-} 1.234}\times {\mathrm{age}}^{\hbox{-} 0.179}\left[\mathrm{if}\ \mathrm{female},\times 0.79\right] $$

In the equation, SCr is the serum creatinine concentration in mg/dL, age is in years.

Serum total cholesterol, triglyceride, and uric acid which were measured in the previous 3 months while in Peking University First Hospital were documented. Results of the monthly hemoglobin A1c review of patients with diabetes were obtained from the hospital’s medical records system.

### Screening strategy

In the cohort study, each participant collected 5 samples labeled as DAY-1, DAY-2, Random, MONTH-2 and MONTH-3, respectively. The gold standard for a persistent albuminuria diagnosis was defined as abnormal ACR for DAY-1, MONTH-2 and MONTH-3 based on the current clinical guidelines [2]. Since single-measurement of ACR is the most common choice for research, and randomized spot urine sample is the most convenient in clinical practice, this study designated DAY-1 Positive and Random Positive as two alternative strategies. Because of the fluctuation of albuminuria, we designated DAY-1 + Random both positive, DAY-1 + DAY-2 both positive, and DAY-1 + Random + DAY-2 all positive as the other three strategies (Fig. 2).

### Statistical analysis

In the cohort study, data were presented as mean ± SD for continuous variables and proportion for categorical variables. Socio-demographic and clinical characteristics were described. The sensitivity, specificity, positive predictive value (PPV), negative predictive value (NPV), and accuracy of each strategy were then calculated using the following formulas:

$$ Sensitivity=\frac{\mathrm{True}\ \mathrm{Positive}}{\mathrm{True}\ \mathrm{Positive}+\mathrm{False}\ \mathrm{Negative}}\times 100\% $$

$$ Specificity=\frac{\mathrm{True}\ \mathrm{Negative}}{\mathrm{True}\ \mathrm{Negative}+\mathrm{False}\ \mathrm{Positive}}\times 100\% $$

$$ Positive\  Predictive\  Value=\frac{\mathrm{True}\ \mathrm{Positive}}{\mathrm{True}\ \mathrm{Positive}+\mathrm{False}\ \mathrm{Positive}} \times 100\% $$

$$ Negative\  Predictive\  Value=\frac{\mathrm{True}\ \mathrm{Negative}}{\mathrm{True}\ \mathrm{Negative}+\mathrm{False}\ \mathrm{Negative}} \times 100\% $$

$$ Accuracy=\frac{\mathrm{True}\ \mathrm{Positive}+\mathrm{True}\ \mathrm{Negative}}{\mathrm{True}\ \mathrm{Positive}+\mathrm{False}\ \mathrm{Negative}+\mathrm{True}\ \mathrm{Negative}+\mathrm{False}\ \mathrm{Positive}}\times 100\% $$

Descriptions of demographic characteristics and validity assessment of the alternative strategies including their sensitivity and specificity were analyzed by IBM SPSS Statistic 20 (IBM Corporation, New York, USA).

### Cost-effectiveness analysis

A cost-effectiveness analysis was conducted based on the cohort study (Fig. 1). Validity assessments of the cohort study revealed that the DAY-1 + DAY-2 strategy, when compared with the DAY-1+ Random strategy, did not show any advantages in sensitivity or specificity. Therefore, the DAY-1 + DAY-2 strategy was excluded from the cost-effectiveness analysis due to the increased time costs associated with it. The gold standard (DAY-1 + MONTH-2 + MONTH-3) was eliminated from the cost-effectiveness analysis for the following reasons: 1) The study initially defined the gold standard for purposes of validity assessment of various strategies and was not concerned with its practicality [8]. Secondly, both the sensitivity and specificity of a gold standard should be 100%. On the perspective of economic evaluation, the strategy, which has no false positive or false negative detection, is incomparable to alternative strategies in cost-effectiveness analysis. Therefore, only DAY-1, Random, DAY-1 + Random, and DAY-1 + Random + DAY-2 strategies were included in the cost-effectiveness analysis.

**Fig. 3:**
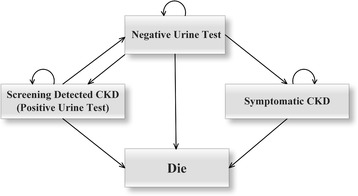
Health States of Markov Model. Negative Urine Test might be true negative to persistent albuminuria of which patient would stay in their present state, or might be false negative which would be retested correctly then turn to Positive Urine Test state, or continued to be miss-diagnosed until symptoms of CKD occurred then turned to symptomatic CKD state. Patients in Positive Urine Test state would be diagnosed as CKD and receive treatment although part of them were false positive. Patients who recovered by proper treatment and the others who were initially misdiagnosed might reverse to the Negative Urine Test state. Death may occur in any health state

The study next conducted a hybrid decision tree and Markov models to simulate the clinical pathway and long-term outcome. The decision tree was comprised of four arms, representing alternative screening strategies DAY-1, Random, DAY-1 + Random, and DAY-1 + Random + DAY-2, respectively. The decision tree adopted prevalence of albuminuria in the general population in China, as well as sensitivity and specificity of each screening strategy to determine how many individuals were in each state of the Markov model [11]. There are four states in Markov model to simulate the pathways of patients’ screening: 1) Negative Urine Test; 2) Screening detected CKD (Positive Urine Test); 3) Symptomatic CKD; 4) Die (Fig. 3). Patients’ negative urine test results might be a true negative to persistent albuminuria which would continue in this state, or might be a false negative which would be identified correctly in the following months or continued being miss-diagnosed until the patient went to hospital because of CKD symptoms. Patients with positive urine test results would be diagnosed as CKD and would receive treatment although part of the test results were false positive. The patients who recovered with proper treatment and those who were initially misdiagnosed might revert to the Negative Urine Test state. The study assumed that death might happen at any health state. Four Markov models were used simultaneously, one for each of the alternative strategies.

With respect to the evaluation of chronicity and the frequency of follow-up for early stage CKD, the length of model cycle was defined as 12 months. The models were run for a time horizon of 30 years. Model results included both clinical and cost effectiveness outcomes. Cost per quality adjusted life year (QALY) was the primary outcome sought.

The assumption was made that patients would accept annual retest according to the current guidelines [2]. If a patient with diabetes, hypertension, or coronary artery disease is with persistent albuminuria also, the diagnosis of CKD is established and prescription of RAS inhibitors is recommended [2]. The study further presumed that all patients with a positive diagnosis of persistent albuminuria received treatment of ACEI/ARB and that the majority of symptomatic CKD was in stage 3 to 5. These patients had to receive and were charged for kidney disease care [20].

All the model inputs are summarized in Table 1. Validity parameters were derived from results of the cohort study. Costs of alternative strategies were calculated by market price [21]. The only available data on Quality-Adjusted of Life Years among Chinese CKD patients was extracted from the study of Wu et al., in which QALYs of CKD patients were evaluated with the baseline QALYs as 1 [20]. Because of the sparse available data, we had to define the background QALYs in present study as 1 although it is not an ideal setting. Mortality was exacted from the China Health and Family Planning Statistical Yearbook 2013 [18]. Treatment strategies and the following outcomes of ACEI/ARB for persistent albuminuria were approximately simulated based on literatures [22–24]. The study allowed for treatment discontinuation in the model due to noncompliance and side effects of ACEI/ARB. It was assumed that most side effects appeared in the first 3 months of treatment and 25% of patients receiving ACEI/ARB would discontinue. Additionally, the study assumed another 2% loss due to the gradual discontinuation caused by noncompliance and that patients who discontinued ACEI/ARB treatment would not restart [25]. The analysis also investigated a model with costs and benefits discounted at 5% [26]. The study assumed non-informative prior distributions for all model parameters.

**Table 1:** Model parameters

Parameters^a^	Values	Range	Distribution	Sources
General Mortality	0.0119	±25%	Log Normal	China Health and Family Planning Statistical Yearbook 2013 [18]
Relative risk of CKD Mortality	1.63	1.5–1.77	Log Normal	Chronic Kidney Disease Prognosis Consortium 2010 [30]
Relative risk reduction of CKD mortality after treatment	0.24	0.08–0.37	Log Normal	Heart Outcomes Prevention Evaluation (HOPE) Study Investigators 2000 [22]
Cost				
DAY-1	24.0	±25%	Gamma	Market price [21]
Random	24.0			
DAY-1+ Random	48.0			
DAY-1+ Random + DAY-2	72.0			
RAS inhibitors	2867.2			
CKD annual cost	34205.0		Gamma	Wu et al.2014 [20]
Quality-Adjusted of Life Years				
CKD	0.899	±0.145	Beta	Wu et al.2014 [20]

^a^Validity parameters of alternative strategies including true/false positive/negative rate were extracted from Table 3

One-way sensitivity analyses were conducted to investigate the impact on cost-effectiveness results from varying key parameters by plus or minus 25% [27]. We varied treatment efficacy by range of 95% confidence interval and varied the cost by plus or minus 25%. The annual discount rate was varied from 0-10%. Probability sensitivity analysis was also conducted.

The cost-effectiveness analysis and sensitivity analysis were conducted using TreeAge Pro 2011 (TreeAge Software, Inc. Massachusetts, USA).

## Results

### Cohort study

Altogether, 160 participants were included in the present study. There were 2 females and 1 male who did not complete the 2 months following because of hysterectomy under general anesthesia, myocardial infarction, and gastrointestinal bleeding, respectively. Their data and test results were then excluded. Therefore, 157 participants aged 45–90 years were included in our final analyses.

Demographic characteristics are summarized in Table 2. The minimum and maximum of baseline ACR tests were 30.3 mg/g and 295.7 mg/g creatinine. All of the patients were in microalbuminuria magnitude at baseline. The baseline ACR of patients who were diagnosed as persistent albuminuria was higher than the baseline of all participants (106.7 (IQR 50.1, 215.9) mg/g creatinine vs. 63.6 (IQR 40.8, 134.0) mg/g creatinine). The average age was 63.4 ± 9.0 years, and 53.5% were males. The percentage with hypertension and diabetes was 79 and 79.6%, respectively. The majority of patients who had hypertension and/or diabetes controlled their blood pressures and/or HbA1c levels well. The proportion of patients with coronary heart disease and stroke were 19.7 and 8%, respectively. Nearly 50% patients were using ARB/ACEI.

**Table 2:** Demographic Characteristics

	All Participants		Persistent Albuminuria	
In Total (n)	157		82	
Age; years(SD)	63.4 (9.0)		62.0 (8.9)	
Male; n (%)	84.0 (53.5)		52.0 (63.4)	
Current Smoker; n (%)	39.0 (24.8)		24.0 (46.2)	
Body-mass Index(kg/m^2^; SD)	24.7 (6.6)		26.1 (3.3)	
Primary Disease; n (%)				
Hypertension	124 (79.0)		66 (80.5)	
Diabetes	125 (79.6)		66 (80.5)	
Others	25 (15.9)		13 (15.9)	
SBP ≤ 140 mmHg	92/124 (74.2)		65/82 (79.3)	
HbA1c ≤ 8%	92/125 (73.6)		59/82 (72.0)	
History; n (%)				
Coronary heart disease	31 (19.7)		17 (20.7)	
Stroke	13 (8.3)		5 (6.1)	
Cancer	19 (12.1)		7 (8.5)	
Laboratory Tests; mean(SD)				
Uric acid(μmol/L)	324.1 (80.6)		335.0 (76.9)	
Triglyceride(mmol/L)	1.6 (1.0)		1.6 (0.9)	
LDL cholesterol(mmol/L)	2.5 (0.8)		2.4 (0.9)	
HDL cholesterol(mmol/L)	1.1 (0.3)		1.1 (0.3)	
Hemoglobin A1c(%)	5.7 (3.3)		5.9 (3.3)	
Creatinine (μmol/L)	92.0 (21.12)		96.0 (22.3)	
eGFR(mL/min per 1.73m^2^)	75.7 (20.6)		73.4 (18.1)	
ACR(mg/g creatinine; median[IQR])	63.6 (40.8–134.0)		106.7 (50.1–215.9)	
Drugs				
ACEI (%)	13 (8.3)		7 (8.5)	
ARB (%)	75 (47.8)		41 (50.0)	
CCB(%)	69 (72.0)	/125 HT	38 (57.6)	/66 HT
Statin(%)	87 (55.4)		47 (57.3)	

### Validity of screening strategies

According to the golden standard, 82 of 157 patients had persistent albuminuria. Table 3 shows sensitivity, specificity, positive predicted value, negative predicted value, and accuracy of the five strategies. All strategies were with sensitivity higher than 90%. The sensitivity of DAY-1 strategy reached 100.0% while its specificity was 69.3%. Random strategy had the lowest specificity of 46.7% and accuracy of 72.6%. Adding another sample taken on DAY-1 to retest, adding a randomized spot specimen on the same day (DAY-1 + Random), or a first morning specimen on the consecutive day (DAY-1 + DAY-2) did not substantially improve the sensitivity or specificity. Validity performances of DAY-1 + Random and DAY-1 + DAY-2 were quite similar to each other. Compared with other strategies, DAY-1 + Random + DAY-2 showed a relatively higher specificity, sensitivity and accuracy, which were 81.3, 93.9 and 87.9%, respectively.

**Table 3:** Performance of screening strategies

	DAY-1	Random	DAY-1 + Random	DAY-1 + DAY-2	DAY-1+ Random + DAY-2
Validity Assessments					
True Positive No.	82	79	79	79	77
Sensitivity %	100	96.34	96.34	96.34	93.9
95%CI	95.6–100.0	89.7–99.2	89.7–99.2	89.7–99.2	86.3–98.0
True Negative No.	52	35	57	58	61
Specificity %	69.3	46. 7	76.0	77.3	81.3
95%CI	57.6–79.5	35.1–58.6	64.7–85.1	66.2–86.2	70.7–89.4
False Positive No.	23	40	18	17	14
False Positive %	30.7	53.3	24.0	22.7	18.7
95%CI	20.5–42.4	41.5–65.0	14.9–35.3	13.8–33.8	10.6–29.3
False Negative No.	0	3	3	3	5
False Negative %	0	3.66	3.66	3.66	6.1
95%CI	0–4.4	0.8–10.3	0.8–10.3	0.8–10.3	2.0–13.7
PPV %	78.1	66.4	81.4	82.3	84.6
NPV %	100.0	92.1	95.0	95.1	92.4
Accuracy %	85.4	72.6	86.6	87.3	87.9
Cost-effectiveness Analysis					
Cost (¥)	5167.42	11063.42	9035.12	–	18652.73
Incremental Cost (¥)	0.00	2028.30	3867.70	–	7589.32
Effectiveness (QALYs)	10.85	11.13	10.88	–	11.87
Incremental Effectiveness (QALYs)	0.00	0.25	0.03	–	0.73
ICER (¥/QALYs)	0.00	8134.69	112335.88	–	10327.99

*PPV* Positive Predict Value, *NPV* Negative Predict Value, *QALY* Quality-Adjusted of Life Year, *ICER* Incremental Cost-effectiveness Ratio

### Cost-effectiveness analysis for screening strategies

Results of the base case analysis of cost-effectiveness are shown in Table 3. DAY-1 + Random + DAY-2 had the highest effectiveness and costs of 11.87 QALYs and ¥18,652.73 per person. Compared with DAY-1, when 1) DAY-1 + Random strategy was applied, costs increased by ¥3867.70 and effectiveness by 0.03 QALYs; 2) When the Random strategy was applied, costs increased by ¥2028.30 and effectiveness by 0.25 QALYs; 3) When the DAY-1 + Random + DAY-2 strategy was applied, costs increased by¥7589.32 and effectiveness by 0.73 QALYs. Model estimates of Incremental Cost-effectiveness Ratios (ICERs) were calculated as ¥112,335.88/QALYs for DAY-1 + Random; ¥8134.69/QALYs for Random and ¥10,327.99/QALYs for DAY-1 + Random + DAY-2. DAY-1 + Random and Random were absolutely dominated by DAY-1 and by DAY-1 + Random + DAY-2.

### Sensitivity analysis

A threshold to judge cost-effectiveness was also drawn, which is ¥100 thousand/QALY (three times gross domestic product (GDP) per capita). One-way sensitivity analysis showed that, compared DAY-1 + Random + DAY-2 to DAY-1 + Random, DAY-1 and Random respectively, variables including false negative rate of DAY-1 + Random + DAY-2, true positive rate of DAY-1 + Random, true positive rate of Random, cost and utility of CKD, impact on incremental cost and effectiveness the most.

At a willingness-to-pay threshold of ¥100,000 per QALY, the probability of each strategy being cost effective was 91% for DAY-1 + Random + DAY-2, 8% for Random, 1% for DAY-1 and 0% for DAY-1 + Random. The DAY-1 + Random + DAY-2 strategy showed absolute superiority to other strategies.

## Discussion

Our study focused on the very beginning step of CKD screening. It revealed, in order to diagnose persistent albuminuria correctly and quickly, and to guide clinical treatment, the strategy of combining two first morning urine samples and one randomized spot urine sample on two consecutive days is accurate, time-saved and cost-effective. Multi-time sample collection of this strategy adjusted for the variation of protein excretion of spot urine samples. It is much more convenient to operate and much easier to control specimen quality than 24 h urine collection, so this procedure could be generalized to both clinical and research settings. Diagnosis and treatment based on single morning or random urine sample might lead to overestimation of prevalence, as well as unnecessary treatment.

Patient compliance is essential in both clinical and research settings. The time window of 2 months suggested by current guidelines is too long to apply in large-scale studies and clinical practice, especially for patients with mild albuminuria and risk factors of CKD. While they are more likely to ignore the follow-up because of the mild to moderate magnitude of albuminuria, these patients are at an extremely high risk of CKD. The present study focused on this group, phoned 1460 candidates who met the inclusion criteria and did not meet exclusion criteria, and invited them to take part in the three time points, two months follow-up study. Only 160 of them were willing to participate. Overlong duration of follow-up was the main reason for their rejection. Many of them indicated that they would like to take part in the 2-day scenario in the first month if it was allowed. In another population-based study in West Malaysia, only 52.2% of respondents with albuminuria agreed to be retested in the following weeks [8]. This research was a sub-study of another large-scale cohort. It is reasonable to deduce that the group who were willing to simultaneously participate in two trials is characterized by better compliance than the general outpatient; however, their response rate was still low. Hence, shortening the diagnosis time window and simplifying the screening process would be effective to improve patient compliance. In doing so, the feasibility of the screening strategy would be improved in both clinical practice and population-based studies.

In addition to the strategy suggested by current clinical guidelines, several alternative strategies were investigated in previous studies, including single randomized urine sample ACR, single first morning urine sample ACR, single spot urine sample ACR plus retest by a 24-h sample UAE [2, 6, 7, 28, 29]. The present cohort study assessed the performance of single randomized as well as the first morning urine sample ACR test. A high false positive rate is the major limitation of the single spot urine sample test. The specificity of DAY-1 and Random were 69.3 and 46.7%, respectively. In principle, measurement by 24-h urine sample should be the most accurate method, because it largely controls impacts of physiological factors including the circadian rhythm. However, due to the cumbersome collecting process, it is problematic to apply the 24-h urine test in large-scale populations and it is difficult to control sample quality. Convenience is the major advantage of spot urine sample. However, un-detectability of the variation of urine albumin excretion limits its reliability. With these considerations, the study designed three spot sample-retest strategies including DAY-1 + Random, DAY-1 + DAY-2 and DAY-1 + Random + DAY-2. Results of the diagnostic assessment demonstrate that retesting is beneficial for the recognition of false positives and improving specificity. In this study, DAY-1 + Random + DAY-2 showed the highest accuracy. It is reasonable to deduce that multi-timing spot sample measurements allows the detection of physiological variations in urine albumin excretion. Thus, with this strategy, more false positive cases were recognized and the specificity of the strategy was improved. Adding another spot sample to the first morning sample (DAY-1 + Random and DAY-1 + DAY-2) also increased the accuracy compared with the single test strategy (DAY-1). However, with both the additional random sample and the first morning sample, there was no significant difference in validity between these two-spot-sample strategies although the veracity of DAY-1 was definitely superior to Random.

Previous cost-effectiveness analyses on CKD screening strategies mainly focused on the population and/or test items [10, 16, 27]. Few studies investigated the scenario of persistent albuminuria diagnosis. This study’s model considered the whole pathway from screening to death and compared different strategies for CKD screening. By the approach of cost-effectiveness analysis, the study evaluated DAY-1 + Random + DAY-2 from a societal perspective and demonstrated that this is a cost-efficient and feasible strategy to generalize to large-scale screening.

The analysis results of this study showed, given similar sensitivity, superior specificity yields the highest cost-effectiveness. Evaluating the DAY-1 + Random + DAY-2 strategy, its higher validity rate led to greater performance on effectiveness, although its time and economic costs were also the highest. However, from the perspective of long-term financial expense, reduction of the false positive rate will maximize the economic value through avoiding over-treatment and waste of resources.

This study has limitations that deserve mention. First, a larger sample size would be helpful to make the validity assessment of alternative strategies more stable. One-way sensitivity analysis showed the veracity of alternative strategies was one of the most important factors which impacted the model. In conclusion, a larger sample size would improve not only the veracity of strategy evaluation, but also the robustness of the Markov model. Second, although patients follow various courses other than the states in this study, it was modeled in this way based on available data and literature. Finally, this study defined incidence of CKD as constant in the models although rates increased with age and were influenced by primary disease and complications. We may overestimate the effectiveness.

## Conclusions

This study established a time-saving, accurate, and cost-effective screening protocol for persistent albuminuria. It explored the protocol (which is the combination of two consecutive morning urine samples and a random urine sample) and also evaluated its economic performance to ascertain whether it could be generalized to a large-scale population. Since the strategy is demonstrably practical and cost-effective, it could be generally applied in both clinical practice and researches.

## Abbreviations

ACR: Albumin to creatinine ratio
CKD: Chronic kidney disease
eGFR: Estimated glomerular filtration rate
ICER: Incremental cost-effectiveness ratio
IQR: Interquartile range
MDRD: Modification of diet in renal disease
QALY: Quality adjusted life year
SCr: Serum creatinine
UAE: Urinary albumin excretion

## References

[1] Liu ZH (2013). Nephrology in china. Nat Rev Nephrol.

[2] Garabed Eknoyan, Norbert Lameire, Kai-Uwe Eckardt, Bertram L Kasiske, Wheeler DC. Kidney Disease: Improving Global Outcomes (KDIGO) CKD Work Group. KDIGO 2012 Clinical Practice Guideline for the Evaluation and Management of Chronic Kidney Disease. Kidney inter., Suppl. 2013;3:1-150.

[3] de Zeeuw D, Remuzzi G, Parving HH, Keane WF, Zhang Z, Shahinfar S, Snapinn S, Cooper ME, Mitch WE, Brenner BM (2004). Albuminuria, a therapeutic target for cardiovascular protection in type 2 diabetic patients with nephropathy. Circulation.

[4] Ruggenenti P, Perna A, Gherardi G, Garini G, Zoccali C, Salvadori M, Scolari F, Schena FP, Remuzzi G (1999). Renoprotective properties of ACE-inhibition in non-diabetic nephropathies with non-nephrotic proteinuria. Lancet.

[5] Farmer AJ, Stevens R, Hirst J, Lung T, Oke J, Clarke P, Glasziou P, Neil A, Dunger D, M Colhoun H (2014). Optimal strategies for identifying kidney disease in diabetes: properties of screening tests, progression of renal dysfunction and impact of treatment - systematic review and modelling of progression and cost-effectiveness. Health Technol Assess.

[6] National Kidney Foundation (2012). KDOQI clinical practice guideline for diabetes and CKD: 2012 update. Am J Kidney Dis.

[7] Johnson DW, Atai E, Chan M, Phoon RK, Scott C, Toussaint ND, Turner GL, Usherwood T, Wiggins KJ. KHA-CARI Guideline: Early chronic kidney disease: Detection, prevention and management. Nephrology. 2013;18(5):340–50. 10.1111/nep.12052 23506545

[8] Hooi LS, Ong LM, Ahmad G, Bavanandan S, Ahmad NA, Naidu BM, Mohamud WN, Yusoff MF (2013). A population-based study measuring the prevalence of chronic kidney disease among adults in West Malaysia. Kidney Int.

[9] Boulware L, Jaar BG, Tarver-Carr ME, Brancati FL, Powe NR (2003). Screening for proteinuria in us adults: a cost-effectiveness analysis. JAMA.

[10] Kondo M, Yamagata K, Hoshi SL, Saito C, Asahi K, Moriyama T, Tsuruya K, Yoshida H, Iseki K, Watanabe T (2012). Cost-effectiveness of chronic kidney disease mass screening test in Japan. Clin Exp Nephrol.

[11] Zhang L, Wang F, Wang L, Wang W, Liu B, Liu J, Chen M, He Q, Liao Y, Yu X (2012). Prevalence of chronic kidney disease in China: a cross-sectional survey. Lancet.

[12] Coresh J, Selvin E, Stevens LA (2007). Prevalence of chronic kidney disease in the united states. JAMA.

[13] Zhang L, Zhang P, Wang F, Zuo L, Zhou Y, Shi Y, Li G, Jiao S, Liu Z, Liang W (2008). Prevalence and factors associated with CKD: a population study from Beijing. Am J Kidney Dis.

[14] Naresh CN, Hayen A, Weening A, Craig JC, Chadban SJ (2013). Day-to-day variability in spot urine albumin-creatinine ratio. Am J Kidney Dis.

[15] Couser WG, Remuzzi G, Mendis S, Tonelli M (2011). The contribution of chronic kidney disease to the global burden of major noncommunicable diseases. Kidney Int.

[16] Komenda P, Ferguson TW, Macdonald K, Rigatto C, Koolage C, Sood MM, Tangri N (2014). Cost-effectiveness of primary screening for CKD: a systematic review. Am J Kidney Dis.

[17] Garg AX, Kiberd BA, Clark WF, Haynes RB, Clase CM (2002). Albuminuria and renal insufficiency prevalence guides population screening: results from the NHANES III. Kidney Int.

[18] China Health and Family Planning Statistical Yearbook 2013 [http://www.nhfpc.gov.cn/ewebeditor/uploadfile/2014/04/20140430131845405.pdf].

[19] Ma YC, Zuo L, Chen JH, Luo Q, Yu XQ, Li Y, Xu JS, Huang SM, Wang LN, Huang W (2006). Modified glomerular filtration rate estimating equation for Chinese patients with chronic kidney disease. J Am Soc Nephrol.

[20] Wu Jingjing YL: The Quality Of Life And Work Ability In Patients With Chronic Kidney Disease In Urban China. In: ISPOR 19th Annual International Meeting. vol. 17: Value in Health; 2014: A142.

[21] Beijing Municipal Commission of Development and Reform [http://www.bjpc.gov.cn/].

[22] Heart Outcomes Prevention Evaluation (HOPE) Study Investigators (2000). Effects of ramipril on cardiovascular and microvascular outcomes in people with diabetes mellitus: results of the HOPE study and MICRO-HOPE substudy. Lancet.

[23] de Zeeuw D, Remuzzi G, Parving HH, Keane WF, Zhang Z, Shahinfar S, Snapinn S, Cooper ME, Mitch WE, Brenner BM (2004). Proteinuria, a target for renoprotection in patients with type 2 diabetic nephropathy: Lessons from RENAAL. Kidney Int.

[24] Chang TI, Shilane D, Brunelli SM, Cheung AK, Chertow GM, Winkelmayer WC (2011). Angiotensin-converting enzyme inhibitors and cardiovascular outcomes in patients on maintenance hemodialysis. Am Heart J.

[25] Golan L, Birkmeyer JD, Welch HG (1999). The cost-effectiveness of treating all patients with type 2 diabetes with angiotensin-converting enzyme inhibitors. Ann Intern Med.

[26] Murray Krahn MSC, Gafni A (1993). Discounting in the economic evaluation of health care interventions. Med Care.

[27] Hoerger TJ, Wittenborn JS, Segel JE, Burrows NR, Imai K, Eggers P, Pavkov ME, Jordan R, Hailpern SM, Schoolwerth AC (2010). A health policy model of CKD: 2. The cost-effectiveness of microalbuminuria screening. Am J Kidney Dis.

[28] Clark WF, Macnab JJ, Sontrop JM, Jain AK, Moist L, Salvadori M, Suri R, Garg AX (2011). Dipstick proteinuria as a screening strategy to identify rapid renal decline. J Am Soc Nephrol.

[29] Witte EC, Lambers Heerspink HJ, de Zeeuw D, Bakker SJ, de Jong PE, Gansevoort R (2009). First morning voids are more reliable than spot urine samples to assess microalbuminuria. J Am Soc Nephrol.

[30] Chronic Kidney Disease Prognosis Consortium (2010). Association of estimated glomerular filtration rate and albuminuria with all-cause and cardiovascular mortality in general population cohorts: a collaborative meta-analysis. Lancet.

